# Understanding the complexity of surgical interventions in rcts: the role of process evaluation in the operating theatre

**DOI:** 10.1186/1745-6215-14-S1-P3

**Published:** 2013-11-29

**Authors:** Natalie Blencowe, Nicola Mills, Jenny Donovan, Jane Blazeby

**Affiliations:** 1 University of Bristol, Bristol, UK; 2 University Hospitals Bristol NHS Foundation Trust, Bristol, UK

## 

Surgical interventions are considered complex because they have multiple components which may act independently or interdependently to influence outcomes. These components need to be defined, described and monitored so that interventions can be accurately replicated. Process evaluations have been successfully used to inform the design and monitoring of other complex healthcare interventions and this study therefore aimed to establish the feasibility of undertaking process evaluation in the operating theatre.

Case studies of surgical interventions were undertaken within an internal pilot RCT comparing the effectiveness of laparoscopic adjustable gastric banding and roux-en-Y gastric bypass for morbid obesity. Case studies involved two components: a)video recording, audiorecording and non-participant observation in theatre, and b)semi-structured interviews with surgical, anaesthetic and nursing staff to explore views of key intervention elements.

Three case studies in two centres have been successfully undertaken, including interviews with seven staff. Obtaining complete datasets was challenging due to the unpredictable nature of the theatre environment (inpatient bed pressures, re-ordering of operating lists, lack of time) and reliance on technology (operative masks and anaesthetic machines substantially reduce the quality of audiorecordings). Video and audiorecordings have been synchronised with observational data using Observer XT10 software. Thematic analysis of interviews is underway and triangulation of findings will help clarify the intervention and identify key components and context.

Process evaluation in the operating theatre is feasible and can be used to establish how surgical interventions should be designed and monitored in main RCTs. Whether this should occur routinely is currently uncertain and requires further investigation.

